# Lipid-Derived Biomarkers as Therapeutic Targets for Chronic Coronary Syndrome and Ischemic Stroke: An Updated Narrative Review

**DOI:** 10.3390/medicina60040561

**Published:** 2024-03-29

**Authors:** Thomas Gabriel Schreiner, Bogdan Emilian Ignat, Cristina Grosu, Alexandru Dan Costache, Maria Magdalena Leon, Florin Mitu

**Affiliations:** 1 Department of Medical Specialties III, Faculty of Medicine, University of Medicine and Pharmacy “Grigore T. Popa”, 700115 Iasi, Romania; 2 Department of Electrical Measurements and Materials, Faculty of Electrical Engineering and Information Technology, Gheorghe Asachi Technical University of Iasi, 700050 Iasi, Romania; 3 First Neurology Clinic, “Prof. Dr. N. Oblu” Clinical Emergency Hospital, 700309 Iasi, Romania; 4 Neurology Department, Clinical Rehabilitation Hospital, 700661 Iasi, Romania; 5 Department of Medical Specialties I, Faculty of Medicine, University of Medicine and Pharmacy “Grigore T. Popa”, 700115 Iasi, Romania; 6 Medical Rehabilitation Department, Clinical Rehabilitation Hospital, 700661 Iasi, Romania

**Keywords:** biomarkers, diagnosis, targeted therapy, chronic coronary syndrome, ischemic stroke

## Abstract

The incidence and prevalence of cardiac and cerebrovascular diseases are constantly increasing, with chronic coronary syndrome and ischemic stroke as the leading causes of morbidity and mortality worldwide. According to current knowledge, the heart–brain axis is more than a theoretical concept, with many common pathophysiological mechanisms involved in the onset and evolution of both coronary and cerebral ischemia. Moreover, the focus is on the prevention and early intervention of risk factors in searching for targeted and personalized medical treatment. In this context, this narrative review aims to offer, in a didactic and practice-oriented manner, an up-to-date overview of the role played by lipid-derived biomarkers (from low-density lipoprotein cholesterol to oxylipin and apolipoproteins) in chronic coronary syndrome and ischemic stroke. Firstly, the authors highlight, via relevant epidemiological data, the significant burden of chronic coronary syndrome and ischemic stroke in the general population, thus explaining the need for updated information on this topic. Subsequently, the most important lipid-derived biomarkers and their multiple roles in the pathogenesis of these two disorders are listed. Currently available and experimental targeted therapies based on these lipid-derived biomarkers are presented in the final part of this paper, representing this manuscript’s original and novel input.

## 1. Introduction

Chronic coronary disease (CCD) and ischemic stroke (IS) are two of the most frequent non-communicable disorders worldwide and leading causes of death [1]. According to recent epidemiological data, cardiovascular diseases, mainly coronary heart disease, are estimated to lead to approximately 4 million deaths in Europe annually, representing almost 45% of the mortality rate in European countries [2]. Regarding IS, the global number of IS-related mortality has increased over the last 30 years, accounting for over 17% of all deaths related to cardiovascular diseases (CVDs) [3].

Concerning figures can also be observed in the case of morbidity related to the two abovementioned pathologies. CCD is closely associated with a multitude of other CVDs in a continuum that significantly increases the disability-adjusted life years (DALYs) of these patients [4]. Similarly, IS poses a significant burden at the individual level as the most relevant neurological disorder, leading to a decrease in quality of life along with an increase in general disability status [5].

The financial burden is another aspect that cannot be overlooked, particularly when considering the growing resources allocated for preventing and treating cardiac and cerebrovascular disorders. Recent data illustrate the multitude of factors that must be included in the correct and complete estimation of total costs, which do not include only the expenses of formal health services [6]. One very recent systematic review showed the heterogeneity of the currently available literature on this topic, trying to systematize the financial burden into several categories, such as primary prevention, acute stroke care, and post-acute and rehabilitation costs [7]. Additionally, informal care costs were estimated at around EUR 1.3 billion per year in Europe alone, while indirect costs, such as post-stroke loss of productivity, generated EUR 12 billion in 2017 [8]. Despite differences between high-, middle-, and low-income countries, the following general tendency was observed: IS- and CVD-related costs remain among the highest compared to other neurological and non-neurological diseases [9].

**Figure 1:**
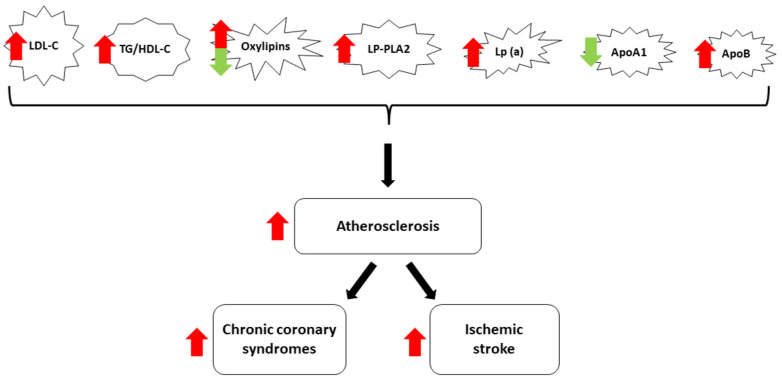
Diagram of the most relevant lipid-derived biomarkers included in this review. Most lipid-derived biomarkers facilitate atherosclerosis and increase the risk for CCS and IS (red arrows). ApoA1 has protective effects (green arrow), while the role of oxylipins is variable.

Considering the abovementioned personal, social, and economic negative impacts of CCD and IS, there is a mandatory need to improve primary and secondary prevention by improving the methods to detect these pathologies as early as possible and by developing sensitive biomarkers correlated with the high risk of subsequent cardiovascular and cerebral diseases. According to current knowledge, atherosclerosis is the primary lipid-related pathological phenomenon linked to both CCD and IS, creating a veritable cardiac–neurologic high-risk profile continuum. In this regard, one natural research direction with promising perspectives is the development and validation of novel lipid-derived biomarkers, useful for both the early diagnosis and monitorization of CCD and IS, with the final purpose of reducing mortality and morbidity. Thus, the main aim of this article is to offer a practically oriented review of the most relevant lipid-derived biomarkers used in daily clinical practice and research. For the first part, the need for continuous research on this particular topic is demonstrated via the latest epidemiological data regarding the impact of CCD and IS. Subsequently, the authors detail the most relevant lipid-derived biomarkers (Figure 1) and their multiple roles in the pathogenesis of these two disorders. Finally, therapeutic approaches targeting these biomarkers are highlighted, and future research directions are also suggested, contributing to the originality and novelty of this manuscript.

## 2. Updated Epidemiological Data

Chronic coronary syndromes (CCSs) are a significant category of the group of coronary artery disease along acute coronary syndromes and include several patient-related clinical scenarios such as ‘stable’ angina, new onset heart failure, and asymptomatic or symptomatic after initial diagnosis [10]. Despite being classified under the umbrella term of CCSs, all these clinical situations have different risks for future cardiovascular events and mortality. The prevalence of stable angina alone is over 10 million people in the United States, with an estimated incidence of over half a million every year [11]. With prevalence increasing with age in both males and females, CCSs are also a relevant part of the CVD spectrum, remaining the leading cause of death worldwide regardless of low, middle, or high-income countries [12]. The lifetime risk for developing CCSs is more significant in men versus women, with up to 50% estimated risk in males in some developed countries [13].

The second leading cause of both disability and mortality worldwide is IS. According to the latest data, the worldwide incidence of IS is over 12 million per year, with a total prevalence of over 100 million and over 6 million stroke-related deaths [14,15]. Moreover, the absolute numbers have increased over the last 30 years, with incident strokes up by 70%, prevalent strokes up by 85%, and deaths from stroke up by 43%. Regarding morbidity, DALYs due to stroke increased by 32%, with substantial variations between low- and high-income populations. According to the latest analysis, mortality and DALY rates are indirectly linked to income, with lower rates in high-income countries [16]. Additionally, projections for the next decade suggest an increase in IS incidence in both sexes, all age groups, and all socioeconomic index populations, meaning an augmentation of the current individual, global, social, and financial negative impact [17].

Considering the worrying figures and clear trends, a multidirectional approach is mandatory. In this context, at least three different directions with promising clinical impact should be addressed in the near future. In the first place, the faster detection of CCS, IS, and related risk factors/comorbidities, ideally from a preclinical or prodromal stage, is needed; secondly, primary prevention measures should be optimized to delay the onset of cerebral and cardiovascular disorders; and finally, an improvement of secondary prevention interventions should slow the evolution of pathological processes and decrease the risk of an acute event repetition. One relatively simple and quantifiable method is using molecular biomarkers for the early detection and monitoring of cerebral and cardiac disorders. There are also some characteristics an ideal biomarker should have, which can be classified into four different categories, as depicted in Table 1 (adapted from the work of Bostan et al. [18]).

Over time, a broad range of molecules have been discussed as potential biomarkers for CCS and IS. However, one category of compounds, namely lipid-derived biomarkers, remains of great interest even nowadays when advanced imagistic and invasive techniques are available. Lipid-derived biomarkers are among the few molecules of interest for CCS pathologies as, with modified levels in IS as well, they are one of the connection points supporting the idea of the brain–heart axis and common pathologies. The atherosclerotic process is one of the critical players sustaining the onset and evolution of both CCS and IS, with lipid plaque as the main structural alteration responsible for the clinical picture. In this regard, with novel data from many recent studies on the abovementioned topic, this review aims to offer an updated overview of the most important lipid-derived biomarkers and their multiple roles in the pathogenesis of CCSs and IS. Furthermore, the manuscript provides insights into the currently available and experimental targeted therapies based on these lipid-derived biomarkers, being not only a theoretical paper but a practically oriented one that can help clinicians make better decisions for the patients.

## 3. Lipid-Derived Biomarkers

### 3.1. Low-Density Lipoprotein Cholesterol

Low-density lipoprotein cholesterol (LDL-C) remains one of the most studied cholesterol fractions, and a relevant marker for many pathologies related to the atherosclerotic phenomenon. Also called “bad” cholesterol, LDL-C facilitates the systemic movement of cholesterol, mainly to the liver and organs where cell repair occurs or the deposition inside vessel walls [19]. As cholesterol is insoluble in water, its association with proteins is mandatory to allow it to flow through systemic circulation; in this regard, the LDL-C particle consists of a monolayer of phospholipid, unesterified cholesterol at the surface membrane, and fatty acid esters of cholesterol forming the hydrophobic core [20]. LDL is produced in the liver and is the result of the two-step conversion of very low-density lipoprotein (VLDL), first metabolized to intermediate-density lipoprotein (IDL) by lipoprotein lipase, which subsequently is converted to LDL by hepatic triglyceride lipase [21]. Another critical player regulating the LDL-C level is the LDL receptor. This receptor consists of a single-chain glycoprotein, is located mainly on the surface of hepatocytes and many other tissues and has the essential role of internalizing LDL cholesterol by forming a ligand–receptor complex through a clathrin-coated pit. Subsequently, receptor-mediated endocytosis and degradation in the lysozyme occur, and this pathway is crucial in maintaining a constant LDL blood level.

Hypercholesterolemia results from excess cholesterol from exogenic or endogenic sources and, most of the time, correlates with increased blood levels of LDL-C. A fasting lipid panel from a peripheral blood sample is currently used in clinical practice worldwide. Determining LDL-C levels is both accessible and well-standardized. According to the latest guidelines, total cholesterol greater than 200 mg/dL and LDL-C greater than 130 mg/dL are considered abnormal [22]. Additional parameters such as fasting glucose, hemoglobin A1c, and the thyroid-stimulating hormone should also be obtained within the same blood sample to rule out secondary causes of hyperlipidemia [23].

Increased LDL-C has become one of the most frequently used biomarkers in IS and CCS because of its many advantages. LDL-C levels should be assessed periodically in the acute and chronic phases of these disorders, as their levels correlate with future prognosis. LDL-C is an established biomarker for increased risk of developing CCS and IS, directly associated with the atherosclerotic phenomenon and the central pathophysiological mechanisms of the two diseases [24]. According to extensive cohort studies [25], increased LDL-C is frequently associated with other modifications suggestive of a metabolic syndrome, such as hypertension, diabetes, and obesity, with the metabolic syndrome being a significant risk factor for CCS and IS [26]. LDL-C is also helpful in the secondary prevention of IS and cardiovascular disorders, being a biomarker for the patient’s adherence to pharmacologic treatment. LDL-C below 70 mg/dL is the current desired target in patients with a medical history of IS [27]. In contrast, in coronary syndromes, the target value is <55 mg/dL according to ESC guidelines and, in recurrent atherosclerotic CVD events within two years, <40 mg/dL.

Regarding CCS, LDL-C also shows multiple reasons for being considered a close-to-ideal biomarker. Oxidation is a crucial step in the formation of atherosclerosis. Since ox-LDL makes up a large portion of foam cells, it is a potent atherosclerosis promoter. Because ox-LDL binds to scavenger receptors more strongly than native LDL, macrophages can internalize it more efficiently, and foam cell production is encouraged. In addition, ox-LDL has several biological characteristics that exacerbate atherosclerosis. These characteristics include stimulating platelet adhesion, cytotoxic effects, growth factor expression enhancement, endothelial dysfunction, and monocyte chemotactic activity. LDL is oxidized mostly by local vascular cells and macrophages in the arterial wall. Research conducted in vitro revealed that even in the absence of oxygen free radicals and processes that produce oxygen radicals, LDL experiences some degree of oxidation during glycation. Due to plasma’s antioxidant qualities, ox-LDL levels are typically low; nevertheless, improvements in ELISA techniques have made it possible to identify these particles with high sensitivity. Research on ox-LDL as a CVD biomarker is becoming increasingly popular due to these developments. Increased circulating levels of ox-LDL were linked to an increased risk of CV events in a meta-analysis comprising 12 trials. Prospective research found a favorable correlation between the ox-LDL/LDL ratio and the Gensini score, which measures the severity of coronary artery disease (CAD) [28]. In addition, patients with carotid artery disease also showed elevated ox-LDL levels. Given the pathophysiological ramifications of this, the theory that ox-LDL may be a more accurate measure of overall CV risk is tenable.

### 3.2. Triglyceride-to-High-Density Lipoprotein Cholesterol Ratio

Triglycerides (TGs) are the main constituents of body fat and represent almost 95% of all dietary fats. TGs constitute a significant component of the sebaceous glands and, by circulating in the blood, enable bidirectional transfer to and from the liver [29]. While the well-known classification of saturated and unsaturated TGs is of interest for medical purposes, TGs alone are not considered a specific biomarker for CVD or IS. Still, their blood level is a frequently monitored parameter in both patients and healthy controls. High-density lipoprotein cholesterol (HDL-C) is another intensely studied fraction of total blood cholesterol, mainly in many studies addressing cardiac and cerebrovascular risk [30]. HDL-C seems to play an atheroprotective role, with several epidemiological studies suggesting its preventive function concerning myocardial infarction, transient ischemic attacks, and stroke [31]. More recent research focused on the functionality of the HDL molecule and its efflux capacity as a potential therapeutic method to enhance the antiatherosclerotic pathways [32]. The TG/HDL-C ratio seems more relevant than TG or HDL-C alone, becoming a more sensitive predictor of CVD risk [33]. The TG/HDL-C ratio could be a linking biomarker between metabolic syndrome and CVD and also a predictor for insulin resistance in obese patients. Several studies examined different cut-off values for this ratio regarding the early detection of metabolic syndrome [34] and the assessment of CVD risk [35]; however, standardized values still need to be determined. The TG/HDL-C ratio is also relevant in detecting type 2 diabetic patients at risk for poor glycemic control, with the values being closely correlated to HbA1c [36].

The TG/HDL-C ratio, closely related to atherosclerosis, has also been intensely studied in coronary artery syndromes, including CCS. The presence and burden of coronary plaques were substantially correlated with the non-HDL-C level and the TG/HDL-C ratio. The non-calcified plaque (NCP) shape was also linked with the serum non-HDL-C level and the TG/HDL-C ratio. Moreover, the TG/HDL-C ratio and non-HDL-C were independent predictors of the non-calcified plaque burden (NCB). They might be employed as low-cost, simple-to-measure statistics to identify young people at high risk of coronary plaques.

Finally, the TG/HDL-C ratio was also linked to cerebrovascular disorders and mainly IS. For example, a Japanese study showed that high TG combined with low HDL-C levels imposed a greater stroke hazard ratio (HR) [37]. The TG/HDL-C ratio was significantly higher in young stroke patients compared to healthy controls [38] and also positively associated with silent brain infarcts [39]. The TG/HDL-C ratio is also relevant post-stroke, as it may predict better prognosis in IS patients. According to one Chinese study, the ratio was independently correlated with a reduced mortality risk [40]. Despite the lack of international guidelines, the TG/HDL-C ratio is a promising biomarker for the prognosis of IS, as it could sensitively predict the risk of having an IS but could also be used during follow-up in the case of IS secondary prophylaxis.

### 3.3. Oxylipin

Oxylipins are oxygenated natural products derived from fatty acids under cyclooxygenase activity [41]. They have recently become of interest to researchers because of their assumed roles in the pathophysiological pathways of several disorders, such as atherosclerosis [42], non-alcoholic fatty liver disease [43], and even Alzheimer’s disease [44]. In humans, this link is explained by the pro- and anti-inflammatory roles of oxylipins, with chronic inflammation being the primary pathological mechanism in the abovementioned noncommunicable diseases. By acting in a paracrine or autocrine manner, oxylipins target peroxisome proliferator-activated receptors (PPARs), finally modifying the formation and function of adipocytes [45].

With several studies linking oxylipins to hypertension, hyperlipidemia, and diabetes, these molecules have gained a potential role as biomarkers in IS and CVD diseases, including CCS. Linoleic acid oxylipins were associated with clinically or imagistically diagnosed cerebral small vessel disease; this finding is consistent with the blood–brain barrier and neurovascular–glial disruptions that occur in vascular neurodegeneration [46]. Oxylipins’ delicate balance, closely related to other structural and functional changes in the case of IS, makes them potential candidates for the early detection of subclinical changes and disease monitoring. The exact mechanism remains to be determined; however, incipient research suggests that the crosstalk between bioactive oxylipins and other molecular pathways was altered in ischemic conditions, such as the unfolded protein response [47]. Additionally, dietary supplementation with anti-inflammatory oxylipins could be a potential therapeutic approach; recent experiments in both animal models [48] and humans [49] suggest the beneficial effect of the long-term administration of plant-derived omega-3 fatty acids in IS treatment and prevention.

Current CAD risk assessment scores, including the 10-year Framingham general CVD risk score, were created with the general public in mind and have demonstrated a lack of effectiveness when managing high-risk CAD in adults. Compared to the 10-year Framingham general CVD risk score, the oxylipin panels improved triple vessel coronary artery disease diagnoses and the survival outlook.

The amount of nutrients and oxygen that cardiomyocytes can use to produce enough energy is limited by CAD, and this problem is worsened when the quantity and severity of coronary artery disease rises or plaque bursts and causes thrombus formation. In this investigation, plasma concentrations of hydroxylated omega-3 polyunsaturated fatty acid (PUFA)-derived epoxides were found to be lower in individuals with a greater level of diseased coronary arteries (≥70% stenosis), specifically in lower levels of 19,20-dihydroxy-docosapentaenoic acid (19,20-DiHDPA). There has never been a report on the relationship between oxylipin concentrations and the number of coronary arteries with this disease. Soluble epoxide hydrolase (sEH), the enzyme that hydroxylates epoxides, is activated by hypoxia and has been suggested as a possible therapeutic target for CAD. It is impossible to rule out the chance that CAD participants were already taking medication, inhibiting soluble cytochrome P450 (CYP450) epoxide hydrolase. The idea that the lower concentrations are a reaction to the hypoxia brought on by arterial occlusions is supported by the steady decline in the concentrations of docosahexaenoic acid (DHA) derivates and 12,13-dihydroxyoctadec-9-enoic acid (12,13-DiHOME) with an increase in the number of sick arteries.

Lower amounts of oxygenated omega-6 PUFA linoleic acid (LA) and arachidonic acid (ARA), specifically hydroxy octadecadienoic acid (HODE) and hydroxy eicosatetraenoic acid (HETE), which are produced either by the oxygenation of lipoxygenases or hydroxylation of CYP1B1, were associated with five-year longevity and not undergoing coronary artery bypass grafting (CABG) surgery. The theory that the increased mid-chain HETE and HODE concentrations are a reaction to the hypoxia brought on by the arterial occlusions is supported by data showing elevated 15-HETE concentrations and lipoxygenase LOX-15 enzymatic activity in hypoxic human cardiomyocytes and cardiac endothelial cells. The atherosclerotic plaque has been found to contain high quantities of HETE, such as 5-HETE, 12-HETE, and 15-HETE, particularly in those that are more likely to burst. Following heart surgery, patients had higher levels of 5-HETE and 12-HETE in their blood. There have been reports of higher 5-HETE, 12-HETE, and 15-HETE concentrations in patients with acute cardiac syndrome. Xu et al. showed elevated circulating concentrations of 5-HETE, 12-HETE, and 15-HETE in CAD patients, while Shishebor et al. and Auguet et al. only reported numerical increases; the latter did not quantify 5-HETE. While less is known about the involvement of mid-chain HODE, the role of high mid-chain HETE in cardiovascular dysfunction has been extensively reported. It has been suggested that one treatment approach for managing CAD is to inhibit the oxygenation step in the LOX pathway. This indicates that the oxylipins, which have been identified as indicators of chronic hypoxia, have clinical significance.

### 3.4. Lipoprotein-Associated Phospholipase A2 (LP-PLA2)

Lipoprotein-associated phospholipase A2 (LP-PLA2), a phospholipase A2 enzyme that catalyzes the cleavage of fatty acids in position 2 of phospholipids, is known to regulate lipid metabolism at the blood level and to mediate vascular inflammation; thus, it is strongly linked to the atherosclerotic process [50]. In plasma, LP-PLA2 circulates mainly bound to LDL and is less associated with HDL, which are the other two essential biomarkers for monitoring cardiac and cerebrovascular disorders. Moreover, the dynamics of LP-PLA2 between LDL and HDL have clinical significance, with variations in these two fractions observed in patients with hyperlipidemia and coronary artery diseases [51]. As LP-PLA2 bound to HDL has antiatherogenic properties, while LDL-bound-LP-PLA2 has the opposite effect, the LP-PLA2 molecule has increasingly relevant roles from both pathophysiological and therapeutical points of view.

In this context of being related to CCS and IS, LP-PLA2 has recently become of interest to researchers. According to recent data, LP-PLA2 could be a valuable complementary biomarker to the current imaging methods in predicting and diagnosing acute IS [52]. The serum levels of LP-PLA2 also correlate with the incidence, severity, and recurrence of IS [53]. Furthermore, LP-PLA2 seems to have a synergic effect with other IS risk factors, such as hypertension and hyperhomocysteinemia [54]. LP-PLA2 could also be used to monitor the outcome of therapeutic measures in acute and chronic settings. One good example is the negative correlation between LP-PLA2 plasma levels and the early recovery of patients after intravenous thrombolysis [55].

LP-PLA2 circulates in the plasma, is mainly linked to low-density lipoproteins (LDLs), and has high selectivity for vascular inflammation. LP-PLA2 contributes to atherosclerosis by hydrolyzing the oxidized phospholipids in the LDL and producing bioactive products like lysophosphatidylcholine, oxidized fatty acids, and arachidonic acid, which can attract monocytes, activate endothelial cells, and stimulate the proliferation of smooth muscle cells. Two techniques are available for quantifying LP-PLA2 as follows: an ELISA immunoassay for mass concentration measurements or a spectrophotometric assay for enzymatic activity assessments. The latter, nevertheless, turned out to be more correct. LP-PLA2 activity has been shown to be a predictor of CV events, including myocardial infarction and CV death, in individuals with stable CAD. LP-PLA2 was positively linked, independent of other CV risk variables, with the presence and severity of CAD in another prospective investigation (*p* < 0.05). Furthermore, hs-CRP and LP-PLA2, when added to a biomarker panel, improved its prediction potential for atherosclerotic carotid disease as determined by ultrasonography.

### 3.5. Lipoprotein (a)

Lipoprotein (a) is a variant of LDL that incorporates apolipoprotein (a) bound to apolipoprotein B100 through a single disulfide bridge. Known as Lp (a), it is one of the established risk factors for atherosclerosis and a common link between IS and CVD [56]. The *LPA* gene remains the main determinant of Lp (a) plasma levels, explaining the wide variations in the general population and the different degrees of correlation to IS [57]. While the optimal range is <14 mg/dL, elevated Lp (a) levels (>50 mg/dL) are associated with increased CVD risk [58]. This could result from the pathogenic properties of Lp (a), which is considered to promote cholesterol deposition in the arterial wall and diminish plasmin formation, thus favoring thrombosis [59].

Due to its significant atherosclerotic role, Lp (a) screening is recommended in high-risk patients (with a personal and family history of premature CVD, recurrent CVD on statin therapy, and familial hypercholesterolemia) [60]. Several studies that correlated the increased plasma Lp (a) with CVDs, including CCS, are additional proof of the importance of this biomarker in patient monitoring [61]. Furthermore, Lp (a) is associated with the severity of CAD, and monitoring this biomarker has implications for the clinical management of CAD patients [62]. By contributing directly to the total levels of LDL, Lp (a) was also studied in relation to other CVD risk factors, such as hypertension. Up until the present, the direct link between Lp (a) and hypertension remains incompletely established; studies in some ethnic groups (hypertensive Afro-Americans) showed markedly elevated Lp (a) levels, while in other populations, no significant differences between normotensive and hypertensive individuals were observed [63]. More recently, a meta-analysis demonstrated the association between high levels of Lp (a) and the risk of IS, particularly the large artery atherosclerosis subtype of IS [64]. Moreover, increased Lp (a) was also associated with unfavorable prognosis and poor functional outcomes in a cohort of IS patients [65].

### 3.6. Apolipoprotein A-I

Apolipoprotein A-I (ApoA1) is the main protein constituent within HDL-C. It plays an essential role in HDL’s size and shape, controls its lipid content, and facilitates cholesterol efflux from peripheral tissues to the liver [66]. Besides its involvement in cholesterol transport, ApoA1 was also demonstrated to inhibit early-stage inflammatory markers, such as adhesion molecules and cytokines, within the atherosclerotic process [67]. Through these mechanisms, ApoA1 was considered a protective factor against atherosclerosis, thus making it a promising biomarker in both CCS and IS and also a potential therapeutic target.

Regarding the implication of ApoA1 in CVD, circulating apoA-I concentrations were considered to be inversely associated with the risk of CAD. Due to the lack of robust evidence sustaining this hypothesis, new studies were recently conducted aiming to clarify this topic. In this context, worth mentioning is a Mendelian randomization study that showed no causality between ApoA1 and CAD [68]. Furthermore, the same study concludes that therapies aiming to increase ApoA1 are unlikely to reduce the risk of heart disease. Another recent meta-analysis showed that, despite promising results in animal models, ApoA1 replacement therapies did not significantly improve arterial atheroma volume in humans [69]. While additional studies should be conducted to clearly state the role of ApoA1 as a biomarker for CVD risk assessment, combining ApoA1 with other markers, such as apolipoprotein B, is another way of obtaining valuable biomarkers, as described later on.

ApoA1 is also of interest in IS patients, with several clinical trials studying this association. Serum ApoA1 levels seem to be lower in IS resulting from atherothrombosis, compared to IS secondary to other mechanisms [70]. Furthermore, ApoA1 levels are dependent on carotid plaque properties, which are another essential factor in IS development. Still, future research should address the question of whether ApoA1 could also be associated with short- and long-term prognosis in IS patients and even if it could be a therapeutic target in IS recurrence prevention.

### 3.7. Apolipoprotein B

Apolipoprotein B (ApoB) is a hydrophobic protein found in cholesterol-carrying lipoproteins, with ApoB100 and ApoB48 as the two isoforms found in circulation. While ApoB100 is synthesized in the liver and serves as the main structural protein on VLDL, ApoB48 is synthesized in the small intestine and is the primary structural protein on chylomicrons. According to the complex lipoprotein metabolism, which is reviewed in detail elsewhere [71], it seems that ApoB levels are an accurate measure of the atherogenic lipoprotein particle number. ApoB provides relevant information on the LDL risk for causing atherogenesis and is strongly associated with CVD [72].

Based on its roles, ApoB has become a valuable molecule for primary and secondary atherosclerosis prevention, strongly linked with CCS and IS pathogenicity. ApoB was proposed as a secondary treatment target to address residual cardiovascular risk and should be preferred as a risk assessment marker over others, particularly in patients with hypertriglyceridemia, obesity, or type II diabetes mellitus. ApoB appears to be more stable and CV risk-related in patients with associated comorbidities than cholesterol, which varies between the different lipoproteins. A recent analysis of 13,015 statin-treated patients found elevated ApoB, and not elevated LDL-C, to be associated with increased all-cause mortality and myocardial infarction [73]. A similar tendency was observed in another cohort, where ApoB was the only biomarker significantly associated with incident myocardial infarction in fully adjusted models [74]. These findings emphasize the potential of ApoB as a more reliable indicator of CCS risk compared to traditional lipid markers. Furthermore, the Apo B/A1 ratio is another relevant derived biomarker that has been demonstrated to be an independent predictor for plaque changes (rupture, erosion, and thrombosis) in patients with atherosclerotic CVD [75].

Similarly, ApoB was discussed in the context of increased IS. An interesting meta-analysis demonstrated a significant association between two *APOB* gene mutations and an increased risk of IS [76]. While studies on the ApoB-IS association are reduced, several trials have analyzed the ApoB/A1 ratio as a potential biomarker for IS. This ratio was an independent predictor for intracranial artery stenosis, which is one of the leading causes of IS [77]. According to an extensive cohort study, ApoB showed the strongest association with large vessel IS and the weakest with cardioembolic stroke [78]. The same study suggested that ApoB and ApoA1, along with their ratio, had a larger magnitude of association with IS compared to lipoprotein cholesterol levels.

In summary, Table 2 highlights the potential roles of lipid-derived biomarkers in diagnosing and the therapeutic monitoring of CCS and IS.

## 4. Therapeutic Approaches Targeting Lipid-Derived Biomarkers

The currently available therapeutic strategies in both IS and CCS are primarily based on symptomatic, older drugs that aim, among other purposes, to lower the total blood lipid level. However, with new lipid-derived molecules having a high chance of becoming the following widely available biomarkers, several targeted therapies could interest clinicians in the coming years. The impact of lifestyle changes is not discussed, as this non-pharmacologic approach is non-specific, leading to a general lowering of all mentioned parameters.

LDL-C is already used as a parameter in the general monitoring of patients with high cardiovascular risk; LDL-C lowering medication is essential in treating cardiac and cerebrovascular pathologies. There are currently eight different classes of cholesterol-lowering drugs: statins, ezetimibe, bile acid sequestrants, PCSK9 inhibitors, bempedoic acid, lomitapide, mipomersen, and evinacumab [79]. According to guidelines, statins are the first line of treatment in the secondary prevention of IS [80]. By inhibiting HMG-CoA reductase, which is an essential enzyme of the mevalonate pathway, statins decrease the hepatic cholesterol content, leading to the up-regulation of hepatic LDL receptors and LDL clearance [81].

Ezetimibe is one of the first alternatives to statins; however, it has less efficacy as it can lower LDL-C levels by only approximately 20% [82]. Ezetimibe’s mechanism of action is based mainly on inhibiting cholesterol absorption at the intestinal level, with a subsequent decrease in the cholesterol delivery to the liver, a reduction in hepatic cholesterol content, and an up-regulation of hepatic LDL receptors. According to current guidelines, ezetimibe is very useful as an add-on therapy and, secondly, is associated with statin therapy in insufficiently controlled patients or in cases of statin intolerance [83].

Bile acid sequestrants indirectly lower LDL-C levels by decreasing the absorption of bile acids at the intestinal level and subsequently stimulating the synthesis of bile acids from cholesterol [84]. However, their use is limited in daily clinical practice because of their adverse effects, as follows: a decrease in the absorption of multiple drugs, a possible increase in triglyceride levels, constipation, and other gastrointestinal side effects [85].

PCSK9 inhibitors are very potent medications, lowering LDL-C levels by 50–60% by inhibiting LDL receptor degradation. Moreover, according to recent results, PCSK9 inhibitors significantly reduced Lp (a) and brought additional benefits to high-risk patients [86]. PCSK9 inhibitors have few side effects and are used with maximally tolerated statin therapy when the LDL target level is not reached [87].

The effect of bempedoic acid on lowering LDL-C results from the inhibition of hepatic ATP citrate lyase activity, which decreases hepatic cholesterol synthesis and causes a decline in the hepatic cholesterol content and the up-regulation of LDL receptors. The recommendations regarding bempedoic acid suggest it be administered when LDL-C goals are not reached in maximally tolerated statin therapy or in cases of statin intolerance. It should be considered that bempedoic acid can be associated with hyperuricemia, gout attacks, and tendon ruptures [88].

Lomitapide is an inhibitor of the microsomal triglyceride transfer protein, thus favoring a decrease in chylomicron formation and hepatic VLDL at the intestinal level [89]. Evinacumab is a monoclonal antibody that inhibits the activity of angiopoietin-like protein 3, subsequently increasing the activity of lipoprotein lipase and endothelial cell lipase [90]. Both drugs are approved as LDL-C-lowering medication in patients diagnosed with homozygous familial hypercholesterolemia as adjunctive therapy. On the other hand, mipomersen, a second-generation apolipoprotein anti-sense oligonucleotide that reduces the formation and synthesis of VLDL, is no longer available because of its potential liver toxicity [91].

Regarding the TG/HDL-C ratio, current therapies aim either to decrease the TG level or increase HDL-C at a systemic level. Despite being highly efficacious in reducing LDL-C levels, statins significantly lower VLDL triglyceride levels according to the already established knowledge [92]. This could seem counterintuitive as statins’ central mechanism of action (increasing the number of LDL receptors) does not suggest a significant effect on other lipoprotein fractions such as VLDL.

Besides statins, three other drug classes are considered appropriate for managing TG elevations as follows: fibric acid derivatives, niacin, and omega-3 fatty acids. Fibric acids are PPAR-alpha agonists, increasing fatty acid oxidation, eliminating triglyceride-rich particles, and increasing the catabolism of VLDL via this mechanism [93]. Despite their effectiveness in reducing plasma TG levels by 30 to 60%, side effects such as gastrointestinal symptoms, elevated liver enzymes, or nonspecific neurologic complaints (headache, dizziness) should always be considered. Niacin, also known as vitamin B3, has shown, in addition to the hypolipidemic function, many other pleiotropic effects, such as antioxidative and anti-inflammatory actions [94]. While niacin was considered to be a powerful drug for the treatment of mixed dyslipidemia, especially in high-risk patients when the target goals of LDL-C are not achieved with statin therapy, recent extensive randomized clinical studies delivered some disappointing results [95]. Omega-3 fatty acids might modulate different pathways, such as the activation of the transcription factor NFE2-like bZIP transcription factor 2 (NFE2L2), the reduction in nuclear factor-kappa B (NFKB), which induces the expression of inflammation-related genes, or the activation of PPARs [96]. All of these explain omega-3 fatty acids antioxidant effect, as well as the reduction in the expression of lipogenic genes, subsequently explaining the TG-reduction effect.

Oxylipins could be a treatment per se, with recent studies suggesting their beneficial role in the generalized lowering of cholesterol and TG. Current research focuses on better understanding several mechanisms modulated by oxylipins. The cellular and molecular pathways are more complicated than expected, as even immune cells such as lymphocytes are involved in oxylipin secretion and subsequent fatty acid level modulation [97].

LP-PLA2 is not a new target when considering hypolipidemic drugs. Because of its tight association with CVD and IS, LP-PLA2 was first considered a valuable statin target more than ten years ago [98]. More specific are, however, the LP-PLA2 inhibitors, which have been intensely explored in recent years. Several classes of inhibitors were studied, including pyrimidone derivates, biaryl inhibitors, lactam inhibitors, sulfonamide inhibitors, and covalent inhibitors [99]. However, except for pyrimidone derivates, particularly darapladib and rilapladib, the other molecules were only sparsely utilized in clinical trials, mainly due to the difficulty in balancing these molecules’ inhibitory potency and physicochemical properties. Future research should focus on the role of genetics and improving the biological functions of LP-PLA2 inhibitors [100].

Table 3 summarizes the currently available and experimental therapeutic options that target the most relevant lipid-derived biomarkers that could effectively treat IS and CCS.

## 5. Future Research Directions

The topic of lipid-derived biomarkers is broadly open to significant changes in the next few years. Considering the recent discoveries in the fields of cardiology and neurology, together with the tremendous research conducted on CCS and IS patients, new sensitive and specific molecules for the earlier detection of these pathologies could be available soon. Moreover, the current biomarkers might suffer modifications regarding the normal and pathological thresholds or their correlation with other CV risk factors, such as hypertension. The benefit of artificial intelligence-based techniques, such as deep learning, is that they could gather the abovementioned lipid-derived biomarkers within more extensive risk assessment profiles in CVD and IS patients. Finally, advancements in the better understanding of the onset and evolution of cardiac and cerebrovascular disorders could increase the importance of these biomarkers in the early prevention and regular monitoring of the disorder. 

Another highly probable aspect in the near future is related to the targeted therapies directed against or symbiotic to the lipid-derived biomarkers. Although multiple lipid-lowering therapies exist, advancements in targeted inhibitors and monoclonal antibodies offer interesting perspectives. By modulating the molecules involved in lipid metabolism directly and specifically, novel therapies can be more efficient, concomitantly, with fewer adverse effects. LP-PLA2 inhibitors and oxylipins are two relevant examples of therapies currently in the phase of clinical studies. Similarly, agents aiming to lower Lp (a), such as pelacarsen or olpasiran, are presently being evaluated in clinical trials and, if proven efficient, could become new targeted cholesterol-lowering therapies [101]. Finally, the therapies that have already been approved should not be forgotten. Despite their demonstrated efficacy, some molecular mechanisms still need to be completely understood, such as the impact of dabigatran on the lowering of ApoB levels. Additionally, reducing medication-related adverse effects should be another priority for future studies.

## 6. Conclusions

Despite the significant number of studies on the prevention and treatment of cardiac and cerebrovascular diseases, their incidence and prevalence are on a constant rise worldwide. CCS and IS, in particular, represent the leading causes of morbidity and mortality regardless of country or region, posing an increasing economic burden. In this global context, there should be an enhanced focus on prevention and early intervention for risk factors, concomitantly with targeted and personalized medical treatment.

In line with the theoretical advancements related to the heart–brain axis, the common pathophysiological mechanisms involved in the onset and evolution of both coronary and cerebral ischemia suggest the new research direction highlighted in this review. Several essential molecules are involved in the pathogenesis of both IS and CCS, with relevant clinical applications. These molecules are potential biomarkers for the early detection of patients at high risk of developing cardiac and cerebrovascular events and also attractive therapeutic targets for currently available and future drugs.

The authors have presented in detail the five most relevant lipid-derived biomarkers currently used by neurologists and cardiologists in daily clinical practice and research. Several shortcomings were highlighted when comparing these molecules with the characteristics of an ideal biomarker. However, when compared to classical risk factors, these modern biomarkers are more precise and correlate better with the burden and stage of the disease. Moreover, they are relevant from a therapeutic point of view, with several classes of lipid-lowering drugs specifically targeting LDL-C, ApoB, and LP-PLA2. Modulating the critical molecules in lipid metabolism via targeted therapies raises treatment efficacy while reducing adverse effects.

In conclusion, there is a continuous need to improve current cardiac and cerebrovascular biomarkers while developing new tools for the early diagnosis of high-risk patients. According to the characteristics of the ideal biomarkers, these molecules should also be valuable targets for personalized therapies that are expected to emerge soon.

## Figures and Tables

**Table 1:** Characteristics of an ideal biomarker for detecting and monitoring CCS and IS.

Characteristics of the Biomarker	Detailed Clinical Situation
High sensitivity	Rapid release that allows fast diagnosis
	Long half-life that allows repeatedly monitoring
	Correlated with disease severity
High specificity	Absent in healthy persons
	Absent in other tissues except the heart and brain
	Absent in differential diagnosis disorders
Assay-related parameters	Cost-effective
	Non-invasive or minimally invasive sample obtaining
	Short processing time
	Standardized method (with clear thresholds)
Clinical setting	Useful for early detection
	Useful for therapy monitoring
	Useful for predictions and prognosis

**Table 2:** Lipid-derived biomarkers for diagnosis and monitoring of CCS and IS.

Lipid-Derived Biomarker	Utility in Chronic Coronary Syndromes	Utility in Ischemic Stroke
Low-density lipoprotein cholesterol	Associated with an increased risk of CV events. Favorable correlation between the ox-LDL/LDL ratio and the Gensini score for CAD	Associated with increased risk of developing IS Helpful in the secondary prevention of IS The main target of cholesterol-lowering drugs
Triglyceride-to-high-density-lipoprotein cholesterol ratio	Associated with the presence and burden of coronary plaque. Independent predictors of the non-calcified plaque burden	Helpful in evaluating the stroke hazard ratio. Correlated with mortality
Oxylipin	Indicator of chronic hypoxia. Potential correlation with the number of affected coronary arteries	Early detection of subclinical changes in IS. Potential treatment for the chronic post-stroke phase
Lipoprotein-associated phospholipase A2	Predictor of CV events such as myocardial infarction and CV death. Predictor for atherosclerotic carotid disease	Complementary biomarker to current imaging methods in predicting and diagnosing acute IS. Helpful in monitoring the outcome of therapeutic measures in acute and chronic IS
Lipoprotein (a)	Associated with the severity of CAD. Potential correlation with other CV risk factors (hypertension). Independent CV risk factor	Associated with increased risk of large artery atherosclerotic IS. Correlated with functional outcome and prognosis
Apolipoprotein A-I	Potential protective factors, When combined with ApoB (ApoB/A1 ratio), it is an independent predictor for plaque modifications	Predictor for atherothrombotic IS. Correlated with carotid plaque phenotype
Apolipoprotein B	More stable biomarkers in evaluating CV risk compared to cholesterol. Significantly associated with incident myocardial infarction	Independent predictor for intracranial artery stenosis. Strong association with large vessel IS

**Table 3:** Therapeutic options targeting lipid-derived biomarkers.

Drug	Molecular Mechanism	Targeted Lipid-Derived Biomarker	Current Clinical Status
Statins	Inhibition of HMG-CoA reductase	Low-density lipoprotein cholesterol Triglyceride-to-high-density-lipoprotein cholesterol ratio Lipoprotein-associated phospholipase A2 Apolipoprotein B	First-line therapy
Ezetimibe	Inhibition of intestinal cholesterol absorption	Low-density lipoprotein cholesterol Apolipoprotein B	Add-on therapy to statins
Bile acid sequestrants	Decrease in bile acid absorption	Low-density lipoprotein cholesterol	Used in combination with niacin and ezetimibe
PCSK9 inhibitors	Inhibition of LDL receptor degradation	Low-density lipoprotein cholesterol Lipoprotein (a) Apolipoprotein B	Adjunct to diet, used alone or combined with other LDL-C lowering therapies
Bempedoic acid	Inhibition of hepatic ATP citrate lyase activity	Low-density lipoprotein cholesterol Apolipoprotein B	Adjunct to maximally tolerated statin therapy
Lomitapide	Inhibition of the microsomal triglyceride transfer protein	Low-density lipoprotein cholesterol Apolipoprotein B	Adjunct to diet, combined with other LDL-C-lowering therapies
Evinacumab	Inhibition of angiopoietin-like protein 3 activity	Low-density lipoprotein cholesterol	Adjunct to other cholesterol-lowering treatments
Mipomersen	Reduction in VLDL formation and synthesis	Low-density lipoprotein cholesterol Apolipoprotein B	Discontinued
Niacin	Modulation of liver synthesis of triglycerides, and limitation of VLDL assembly	Triglyceride-to-high-density-lipoprotein cholesterol ratio Lipoprotein (a) Apolipoprotein B	Adjunct to other cholesterol-lowering treatments
Fibric acid derivates	Increasing fatty acid oxidation, triglyceride-rich particle elimination, and VLDL catabolism	Triglyceride-to-high-density-lipoprotein cholesterol ratio Apolipoprotein B	Adjunct to other cholesterol-lowering treatments
Omega 3 fatty acids	Reduction in lipogenic gene expression	Triglyceride-to-high-density-lipoprotein cholesterol ratio	Adjunct to other cholesterol-lowering treatments
Dabigatran	Binding competitively to the active site on human thrombin Pleiotropic effect of ApoB-lowering	Apolipoprotein B	Not primarily used ApoB-lowering medication
LP-PLA2 inhibitors	Inhibition of LP-PLA2	Lipoprotein-associated phospholipase A2	Clinical studies
Oxylipins	Modulation of pro- and anti-inflammatory pathways	-	Clinical studies

## Data Availability

All data generated or analyzed during this study are included in the published article.
